# The prevalence of factor VIII and IX inhibitors among Saudi patients with hemophilia: Results from the Saudi national hemophilia screening program

**DOI:** 10.1097/MD.0000000000005456

**Published:** 2017-01-13

**Authors:** Tarek Owaidah, Abdulkareem Al Momen, Hazzaa Alzahrani, Abdulrahman Almusa, Fawaz Alkasim, Ahmed Tarawah, Randa Al Nouno, Fatima Al Batniji, Fahad Alothman, Ali Alomari, Saud Abu-Herbish, Mahmoud Abu-Riash, Khawar Siddiqui, Mansor Ahmed, SY Mohamed, Mahasen Saleh

**Affiliations:** a Department of Pathology and Laboratory Medicine, King Faisal Specialist Hospital and Research Center; b Center of Excellence in Thrombosis and Hemostasis, King Saud University; c Oncology Center; d Department of Pediatric Hematology, King Faisal Specialist Hospital and Research Center; e Department of Pediatric Hematology, Ministry of Health, Riyadh; f Department of Pediatric Hematology, Ministry of Health, Medina; g Department of Pediatric Hematology, Military Hospital; h Department of Pediatric Hematology, Security Force Hospital; i Department of Pediatric Hematology, National Guard Hospital; j Department of Oncology, Security Forces Hospital, Riyadh, Saudi Arabia.

**Keywords:** factor inhibitors, factor IX, factor VIII, hemophilia, hemostasis, Saudi Arabia

## Abstract

Hemophilia A and B are X-linked diseases that predominantly affect male patients. Patients can develop coagulation factor inhibitors, which exponentially increases the treatment cost. However, the prevalence of factor VIII and IX inhibitors in Saudi Arabia is unclear.

This study aimed to determine the Saudi prevalence of factor VIII and IX inhibitors.

This 4-year, 7-center, cross-sectional study evaluated the Saudi prevalences of hemophilia A and B. We collected the patients’ clinical data, evaluated their disease, and tested for factor inhibitors.

We included 202 patients with hemophilia (median age at diagnosis: 0.13 years, range: birth–34.8 years). The patients included 198 male patients (98%), 148 patients with hemophilia A (73.3%), and 54 patients with hemophilia B (26.7%). The patients exhibited severe factor VIII activity (<1%; 121 patients; 5.2%), moderate activity (1–5%; 7 patients; 4.9%), and mild activity (14 patients; 9.9%). Among the patients with care-related data, most patients were treated for episodic bleeding (76.8%) or received prophylaxis (22.6%); 1 patient received both treatments. Among the patients with source-related data, the factor replacements were derived from plasma (48.4%), recombinant concentrates (22.9%), both sources (14.6%), or fresh frozen plasma (14.1%). Factor VIII inhibitors were observed in 43 (29.3%) of the 147 patients, and only 1 of the 54 patients developed factor IX inhibitors. Most patients who developed inhibitors had severe hemophilia (40/44; 90.9%), and inhibitors were also common among patients who received recombinant products (14/43; 32.6%).

The Saudi prevalence of factor inhibitors was similar to those among other ethnic populations.

## 1 Introduction

Hemophilia is a bleeding disorder that is caused by X-linked genetic alterations in the production of coagulation factors, which are important for maintaining hemostasis. The most common type is hemophilia A, which involves factor VIII (FVIII) deficiency and affects male patients at a prevalence of 1 : 5000 to 10,000. Hemophilia B involves factor IX (FIX) deficiency, and its prevalence is approximately 1 : 34,500 male patients.^[1]^ Although both disorders are rare, they can be life threatening and expensive to treat, as they require constant replacement of the deficient factor. There are 2 types of factor concentrates (plasma-derived factors and recombinant factors), which are associated with varying rates of inhibitor formation. The development of inhibitors is the most serious complication of hemophilia treatment, and creates an enormous economic burden.^[2]^ These inhibitors are usually classified according to their plasma levels as “high-titer” inhibitors [activity of >5 Bethesda units (BUs)/mL] or “low-titer” inhibitors (<5 BU/mL), although some patients develop transient inhibitors (usually low-titer inhibitors that never exceed 5 BU/mL and disappear spontaneously over time).^[3–6]^ Many high-responder patients will exhibit inhibitor titers that resolve to low or undetectable levels after abstinence from FVIII treatment.

The risk factors for inhibitor development can be patient-related factors (e.g., genetic, ethnic, or immune factors) or treatment-related factors (e.g., type of product used, age at the first treatment/exposure, and treatment duration and intensity).^[7–12]^ Major histocompatibility complex II polymorphisms and other immune mediators may also affect inhibitor development.^[13]^ The presence of inhibitors has major effects on bleeding control, arthropathy status, and quality of life. Unfortunately, severe hemophilia cases become more resistant to the replacement therapy and require high doses of factor replacement to control their bleeding symptoms.^[4]^ The reported prevalences of factor inhibitors are ≤30% among patients with hemophilia A and ≤5% among patients with hemophilia B.^[4,7,9–12]^ Early studies consistently reported that the prevalences of inhibitors were 25% to 32%, although the prevalence may be as low as 12%, because some antibodies disappear over time. Ethnicity affects inhibitor development, as African-American and Latino patients with hemophilia A have a 2-fold higher prevalence of inhibitors, than Caucasian patients with hemophilia A. Nevertheless, there are few reports regarding the prevalence of inhibitors in populations from the Eastern Mediterranean region (e.g., Arabs). Therefore, the present study was performed to provide the first evaluation of FVIII and FIX inhibitors in Saudi Arabia.

## 2 Methods

### 2.1 Design

This cross-sectional screening study involved 7 centers from the central and western regions of Saudi Arabia, and evaluated patients from May 2008 to December 2011. Each center treated patients with hemophilia using replacement therapy, and had the ability to perform factor testing (either on-site or at another tertiary care facility). All patients underwent a clinical examination, blood testing, and a short standardized survey to collect their demographic and clinical data. This survey was performed before the implementation of national guidelines, and the treatments were based on physician experience and the availability of factor concentrates, especially in the rural and remote areas of our country.

The institutional review boards at each center reviewed and approved this study's design, and a representative from each center was invited to participate as a member of the steering committee, which met once or twice each year to review the study's progress and data. All patients provided their informed consent for participation and testing, and their demographic and laboratory data were stored in a password-protected repository.

### 2.2 Patient recruitment and inclusion criteria

The patients were recruited at the centers’ hemophilia clinics and during several hemophilia awareness days. Patients with a diagnosis of congenital hemophilia A or B were included if they fulfilled the following criteria:

1. Factor use for ≥1 year after the first exposure.
2. A diagnosis that was confirmed by the accredited central testing laboratory.
3. Available data regarding the source of the factor replacement.
4. Informed consent from the patient/guardian for participation and blood testing.

### 2.3 Clinical evaluation and blood testing

All patients were assessed to determine their age at diagnosis, type of factor replacement, bleeding history, and joint or organ with recurrent bleeding. The laboratory testing included a complete blood count, partial thromboplastin time, and serological results [hepatitis B virus, hepatitis C virus (HCV), and human immunodeficiency virus (HIV)]. For the blood testing, a 10-mL sample was collected into 3.2% sodium citrate and then centrifuged. The separated plasma samples were transferred to the central laboratory (in Riyadh, Saudi Arabia). Samples that were collected outside of Riyadh were frozen at –70°C, and then shipped to the central laboratory in dry ice.

### 2.4 Coagulation factor and inhibitor testing

The patients’ blood samples were also tested at the central laboratory to determine their FVIII and FIX levels and confirm their diagnosis. In some cases, von Willebrand factor testing was performed to exclude cases that were labeled as FVIII deficiency. All patients underwent chromogenic testing for FVIII:C, FIX, von Willebrand antigen, and ristocetin cofactor activity using the STA-R system and reagents (Stago, Asnieres, France). The normal ranges for FVIII and FIX levels were 50 to 150 IU/dL.

FVIII and FIX inhibitors were measured at the central laboratory using the modified Nijmegen–Bethesda method.^[6]^ Cases with factor inhibitors were classified as mild (<1 BU/mL), moderate (1–5 BU/mL), and severe (>5 BU/mL).

### 2.5 Statistical analysis

The patients’ data were collected using a case report form and entered into a Microsoft Excel spreadsheet (2010); these data were then reviewed by the study coordinators. After the coordinators had confirmed that the dataset was completed, the data were analyzed using SPSS software (version 20; SPSS Inc., Chicago, IL). Continuous data were evaluated using the Shapiro–Wilk test, based on the assumption of a normal distribution. Categorical data were evaluated using Fisher exact test.

## 3 Results

**Figure 1:**
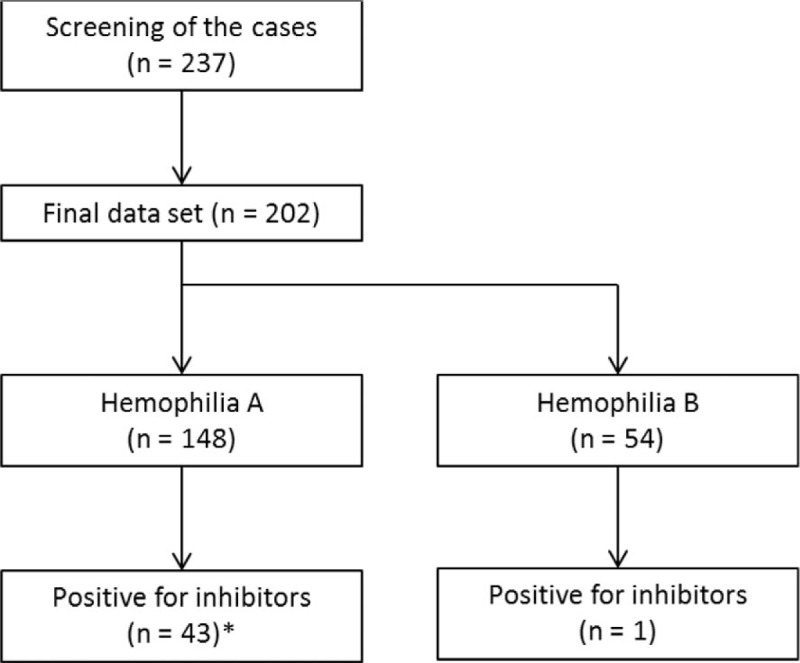
Patient selection and grouping.

A total of 237 patients were enrolled in the study, although we excluded 18 patients for having an incorrect diagnosis (mostly von Willebrand disease), 16 patients for incomplete laboratory data, and 1 patient for unconfirmed inhibitor results. Among the 202 patients with a confirmed diagnosis of hemophilia (Fig. 1), 198 patients were male (98%) and the median age at diagnosis was 0.13 years (range: birth–34.8 years). Most patients (74.9%) were diagnosed before their first birthday. Hemophilia A was observed in 148 cases (73.3%) and hemophilia B was observed in 54 cases (26.7%). There were no significant differences in the age groups according to the type of hemophilia (*P* = 0.892) (Table 1). Most patients with hemophilia A had the severe form (126 patients; 85.7%). A large proportion of the patients with severe disease were diagnosed during infancy (96 patients; 79.3%; *P* = 0.003). Most patients with hemophilia B had the severe form (34 patients; 63%), and most of these patients were diagnosed before the age of 1 year (22 patients; 75.9%) (Table 2).

**Table 1:**
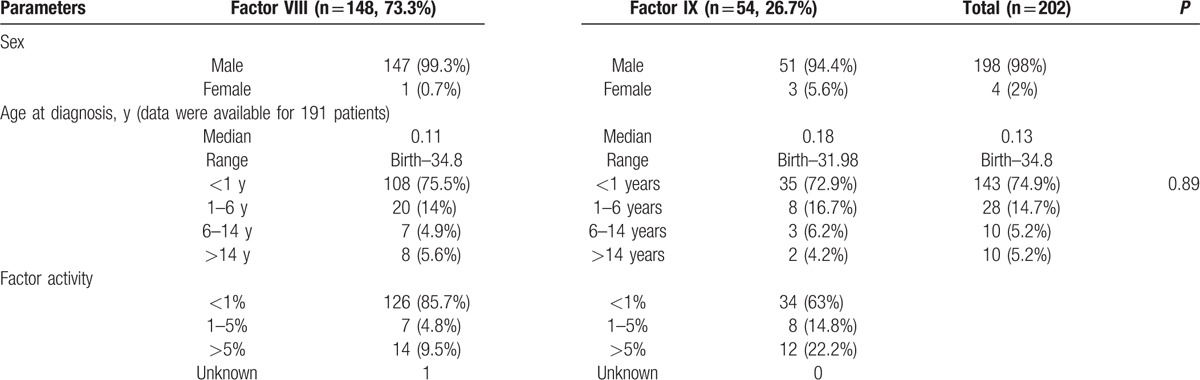
Patients characteristics and disease manifestations.

**Table 2:**
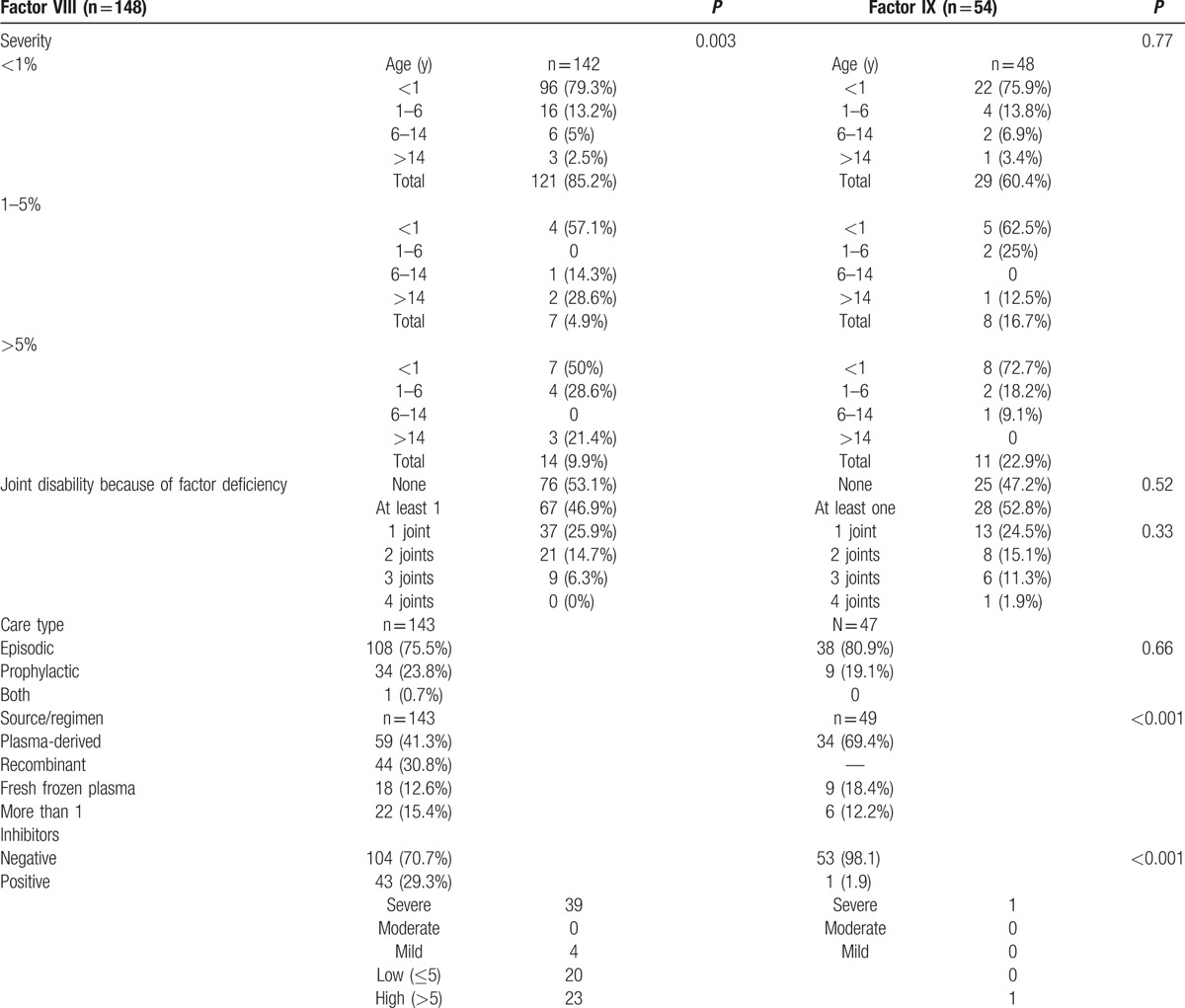
Factor activity, care type, and inhibitor development.

### 3.1 Chronic joint disability

Chronic joint disability because of factor deficiency was observed in 95 of the 196 patients with reliable information (48.5%). Sixty-seven (70.5%) of these patients had FVIII deficiency, and the remaining patients had FIX deficiency. The type of factor deficiency was not significantly associated with joint disability (*P* = 0.521) or number of affected joints (*P* = 0.331) (Table 2). The frequency and number of simultaneously affected joints were higher among patients with FVIII deficiency, although this relationship was not statistically significant (*P* = 0.709) (Table 3).

**Table 3:**
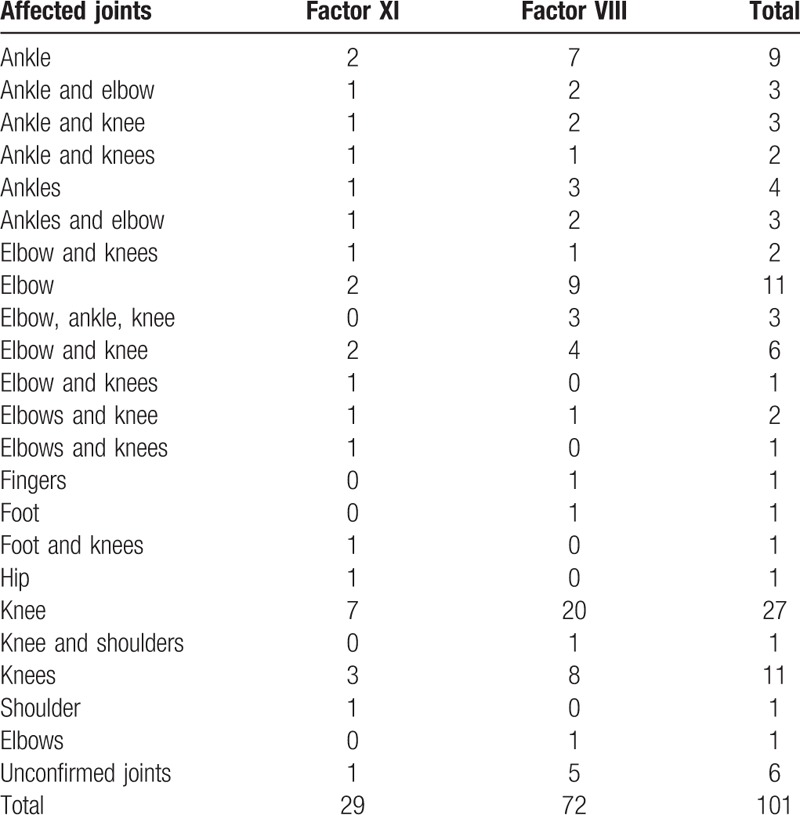
Chronically affected joints (*P* = 0.71).

### 3.2 Care type

Data regarding care type were available for 190 cases. Most patients (146 patients; 76.8%) received treatment for episodic bleeding, and the remaining 44 patients (23.2%) received prophylaxis. Most patients with hemophilia A received episodic treatment (108 patients; 75.5%), although 34 patients (23.8%) received prophylaxis and 1 patient (0.7%) received more than 1 type of product. Most patients with hemophilia B received episodic treatment (38 patients; 80.9%), although 9 patients (19.1%) received prophylaxis (Table 2).

### 3.3 Sources of factor replacement

Information regarding the source of factor replacement was available for 192 patients. The choice of factor replacement was based on the availability of factor concentrates or plasma products at the treatment center. There were no differences in the use of factor concentrates for the hemophilia A and B groups. Ninety-three patients (48.4%) received plasma-derived factors, 44 patients (22.9%) received recombinant concentrate, 28 patients (14.6%) received both, and 27 patients (14.1%) received fresh frozen plasma. During the early study period (2008–2010), the most common source was plasma-derived factors. However, recombinant concentrate was more common during the latter part of the study (2011) (*P* < 0.001).

### 3.4 Inhibitor prevalence and levels

Among the 148 patients with FVIII deficiency, we were able to confirm the inhibitor data for 147 patients. Forty-three patients (29.3%) developed FVIII inhibitors. Twenty patients (46.5%) were low responders (inhibitor titers of >5 BU/mL) and 23 patients (53.5%) were high responders (inhibitor titers of <5 BU/mL). Among the 54 patients with FIX deficiency, only 1 patient developed inhibitors (135.5 BU/mL). This patient was a high-responder male infant with severe disease and 3 affected joints.

Among patients with FVIII deficiency, inhibitor development was most common in the recombinant subgroup (14/43; 32.6%), which was followed by the plasma-derived subgroup (19/59; 32.2%), the group with multiple products (6/22; 27.3%), and the fresh frozen plasma group (4/18; 22.2%). However, these differences were not statistically significant (*P* = 0.883). Inhibitor development was more common among patients who received episodic treatment (34/108; 31.5%), than the prophylactic group (8/33; 24.2%; *P* = 0.247). Inhibitor development was more common among patients with severe disease (39/127; 30.7%), compared to patients with mild disease (4/14; 28.6%; *P* = 0.319).

### 3.5 Viral infection and inhibitor development

Among the 148 patients with FVIII deficiency, reliable data regarding the hepatitis B surface antigen (HBsAg) were available for 105 patients. There was only 1 HBsAg-positive patient and he did not develop factor inhibitors; that patient received fresh frozen plasma. Twenty-eight patients (26.7%) were positive for HCV, and 6 of these patients (21.4%) developed factor inhibitors. Eleven HCV-positive patients (39.4%) received plasma-derived factors, and 8 patients (28.6%) received fresh frozen plasma. Five of the 99 tested patients with FVIII deficiency were positive for HIV (5.1%), and only 1 HIV-positive patient developed inhibitors (Table 4).

**Table 4:**
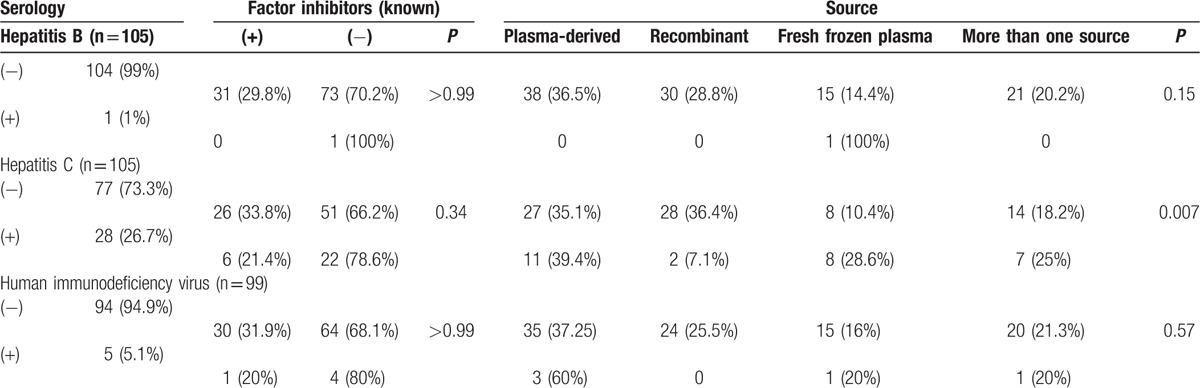
Virus serology, inhibitor development, and source for factor VIII deficiency (n = 148).

Among the 54 patients with FIX deficiency, 2 of the 38 tested patients (5.3%) were positive for HBsAg. Neither of these patients developed factor inhibitors, 1 patient received plasma-derived factors, and 1 patient received multiple products. Data regarding HIV testing were available for 35 patients with FIX deficiency, although none of these patients were HIV-positive.

## 4 Discussion

The management of hemophilia in Saudi Arabia is evolving with changes in the regional and national health care systems. Recent changes in these systems include a nearly complete transition to using recombinant FVIII and FIX; between 2005 and 2014, recombinant factor use increased from 22% to 98% (unpublished data from the Saudi Ministry of Health). Thus, the detection of inhibitors during the study period (May 2008–December 2011) may have been affected by these changes, and our findings should be interpreted with caution. However, most of our findings are likely related to the trends that were established in the years before this study was performed. Furthermore, it is possible that inhibitor development is underreported because of various reasons,^[14]^ such as physician apathy,^[15]^ lack of knowledge that this event should be reported as an adverse reaction,^[16]^ or delayed referral or nonreferral to tertiary hospitals with hematology facilities. These issues are further complicated by the absence of a national registry in Saudi Arabia, the absence of treatment guidelines for small cities, and the absence of recommended testing to detect inhibitor development. Moreover, a study of American patients with severe hemophilia found that only 46% of patients underwent inhibitor testing at hemophilia treatment centers during 2006 to 2010.^[15]^ Voluntary post-licensure reporting can periodically indicate that certain products may be immunogenic.^[17]^ However, the notorious under-reporting of adverse reactions in pharmacovigilance systems suggests that national or even international prospective surveillance databases are needed to provide a sensitive and accurate warning regarding immunogenic products.^[18,19]^

The present study is the first step in a plan to develop a comprehensive understanding of the Saudi phenotypes of hemophilia, replacement therapy use, inhibitor development, and genotypes. Unfortunately, the hemophilia B group only included a small number of patients who developed inhibitors, although this low incidence is consistent with the findings from many international studies. The Centers for Disease Control's Universal Data Collection project during 1998 to 2011 found a low incidence of inhibitors (2%) among 3785 male patients with hemophilia B, although the risk was higher among patients with severe disease [odds ratio (OR): 13.1], patients who were black (OR: 2.2), and patients who were <11 years old (OR: 2.5).^[20]^ Furthermore, many international studies have attempted to link the rate of inhibitor development with ethnicity, factor type and source, care type (prophylactic vs episodic), and genetic or immune characteristics.^[21–23]^ Approximately 29.3% of the hemophilia A group developed inhibitors in the present study, and although approximately one-half of the inhibitor-positive patients had high titers, more than one-quarter of inhibitor-positive patients had low titers and no clinical effects. A prospective follow-up survey of patients with low-titer or transient inhibitors is warranted, as these patients should not be labeled as having hemophilia with inhibitors, which would automatically shift their treatment to a bypass treatment. A more controversial group of patients is the minority (5.4%) of our patients who exhibited intermediate titers (1–5 BU), and further studies are needed to gather more information regarding this population and the properties of their inhibitors.

The incidence of inhibitor formation varies according to ethnicity, with higher rates observed among African-American, Latino, and Hispanic patients, than that among Caucasian patients.^[24,25]^ Western studies typically report a relatively low incidence of clinically significant long-term inhibitors (approximately 10%), although higher rates have been observed when both transient and persistent inhibitors are considered.^[26–30]^ In a recent Japanese study, 26.8% of patients with hemophilia A developed inhibitors, although 70.7% of these patients lost their inhibitors by the end of the 2-year study period (2008–2009).^[30]^ However, there are few studies regarding the prevalence of hemophilia inhibitors among Arab patients. In an Egyptian study, inhibitors were detected in 18.2% of the patients with hemophilia A and in 9.1% of the patients with hemophilia B, and although mild-to-moderate hemophilia was more common than severe hemophilia, inhibitors were more common in patients with severe hemophilia.^[31]^ In a Tunisian study, the prevalence of FVIII and FIX inhibitors was much lower (5%).^[32]^ Pakistan is another Eastern Mediterranean country, although 1 study found inhibitors in only 15% of 140 patients with hemophilia A; these patients exhibited various degrees of severity and different replacement treatments (FVIII concentrate, fresh frozen plasma, or cryoprecipitate).^[33]^ Nevertheless, these discrepancies may be related to differences in the study populations, management trends, and testing strategies. A study of 102 Iranian patients with hemophilia A (44 severe cases, 28 intermediate cases, and 30 mild cases) found that only 20 patients (19.6%) had inhibitors (11 severe cases, 5 intermediate cases, and 4 mild cases)^[34]^; these findings are similar to our present findings. A large Indian study of 1285 patients with hemophilia A found that only 6.07% of the patients had inhibitors, although there were remarkable regional variations (the highest prevalence was 20.99%).^[35]^ We also found a higher prevalence of inhibitors among patients who were receiving recombinant factors, and this result agrees with the findings from our previous studies.^[36–38]^

Although it was difficult to collect comprehensive data regarding treatment intensity in the present study, we found no significant difference in the incidence of inhibitors according to the replacement method (episodic: 21.8%; prophylactic: 21.2%). However, that finding is not universal, and this discrepancy may be related to differences in the protocols for “on-demand” treatment or in the study populations, as differences in the patient population and study design can dramatically alter the findings, even when the same product is evaluated.^[39–41]^ Nevertheless, it was unfortunate that 30% of the patients with hemophilia A became HCV-positive (plasma-derived products: 53%; fresh frozen plasma: 25%; multiple sources: 22%). The acquisition of HCV was likely related to these patients’ cumulative exposures to blood products, as well as their increased risk of surgery, hospitalization, and blood transfusions for bleeding. Although these factors may increase the risk of acquiring HCV, recent changes in the national health care system will hopefully reduce these risks (especially for patients with hemophilia). However, there was no evidence that HCV status affected inhibitor development.

We found that the incidence of FVIII inhibitors was low among HIV-positive patients with severe hemophilia A. A British study also found a low incidence of inhibitors (4.1%) among HIV-positive patients with severe hemophilia, compared with a 3-fold higher incidence among HIV-negative patients (*P* < 0.001), which may be related to the immune status of HIV-positive patients. A Saudi genotype study of contemporary hemophilia A may help define the relationship between inhibitor development and genotype, as disruptive mutations (e.g., intron 22 inversions, large gene deletions, and stop codons) are associated with inhibitor formation in approximately 35% of patients with severe hemophilia A. In contrast, inhibitor formation is observed in as little as 5% of patients with missense mutations or small deletions.^[42,43]^

In conclusion, the economic and health burdens of hemophilia are significant, and inhibitor development can further exaggerate these burdens. Thus, given that we found that inhibitor development was not uncommon in Saudi Arabia, Saudi hematologists should practice prospective pharmacovigilance to follow and report patients who develop inhibitors. Furthermore, a national screening and counseling program for carriers, symptomatic patients, and asymptomatic persons will facilitate the early identification of cases and better management of patients with inhibitors.

## Acknowledgment

The authors thank Novo Nordisk for providing grant support to the National Hemophilia Inhibitor Screening Program.
